# Creative crossroads: advancing sport-event tourism through creative placemaking for enhanced destination value and social impact

**DOI:** 10.3389/fspor.2026.1813176

**Published:** 2026-04-21

**Authors:** Nigina Khalimova, Nutfillo Ibragimov

**Affiliations:** Department of Tourism and Hotel Management, Bukhara State University, Bukhara, Uzbekistan

**Keywords:** co-creation, creative placemaking, creative tourism, destination value, social inclusion, sport-event tourism, sustainable tourism

## Introduction

Sport-event tourism has evolved into a force of considerable economic and cultural importance, shaping destination identities and generating substantial global visitor flows (1, 2). Despite this prominence, the field faces persistent critiques: economic impact assessments frequently overstate net benefits, communities are displaced by event-driven development, cultural assets are commodified for external audiences, and post-event facilities are chronically underutilized (3, 4). These challenges have intensified pressure on event organizers, host governments, and national tourism boards to demonstrate broader social and cultural value.

Creative tourism has emerged as a promising but underutilized response to these challenges. Defined in this article as a form of tourism in which visitors participate in active learning experiences, artistic practices, and authentic cultural engagement that connect them with local communities and creative processes (5, 6), creative tourism prioritizes co-creation—understood here as the collaborative production of tourism experiences through the active participation of both visitors and host communities (7)—as a mechanism for generating authentic, inclusive, and economically diversified destination experiences. The emergence of creative tourism has been further supported by international recognition of the creative economy's role in sustainable development (8, 9).

Despite the apparent complementarity of these fields, their intersection remains surprisingly understudied. A scoping search conducted across Web of Science, Scopus, and Google Scholar using the search strings ‘sport tourism AND creative tourism,’ ‘event tourism AND creative placemaking,’ and ‘sport events AND co-creation’ identified fewer than 15 peer-reviewed articles directly addressing their intersection. This empirical gap justifies the present integrative review at this moment in the field's development: sport-event tourism has reached a scale at which its social and cultural consequences are impossible to ignore, while creative tourism has accumulated sufficient theoretical and practical maturity to offer actionable integration pathways. A framework that bridges these fields is both timely and necessary.

Two concepts are foundational to this article. Creative placemaking refers to the strategic integration of arts and cultural activities into the physical and social fabric of a community to foster civic engagement, cultural vitality, and a distinctive sense of place (10). Destination value refers to the composite of economic, experiential, cultural, and social benefits accruing to a destination as a result of tourism activity, encompassing enhanced visitor spending, improved place reputation, stronger community identity, and increased resident wellbeing (11). Social inclusion, a third key concept, denotes the active participation of diverse community groups—particularly marginalized populations—in the planning, delivery, and benefits of tourism activity, measurable through indicators such as community participation rates, equitable income distribution across local creative enterprises, and resident satisfaction with event-related programming.

This article introduces the Creative Sport-Event Tourism Nexus (CSETN) as an integrative conceptual framework that maps the mechanisms through which creative tourism concepts can be systematically deployed within sport-event tourism contexts. The CSETN is presented as an opinion-based conceptual contribution rather than a fully tested empirical model, consistent with the tradition of framework articles in emerging research domains (12).

### Methodology

This article follows an integrative narrative review methodology (13, 14), which is particularly appropriate for emerging research areas where integration of disparate literatures can generate new theoretical perspectives. A systematic search was conducted across Web of Science, Scopus, and Google Scholar for publications from 2000 to 2024. Search terms included combinations of ‘sport tourism,’ ‘event tourism,’ ‘creative tourism,’ ‘creative placemaking,’ ‘event leveraging,’ ‘co-creation,’ and ‘destination value.’ Inclusion criteria required: (a) peer-reviewed articles or scholarly book chapters; (b) direct relevance to at least one of the five thematic clusters described below; and (c) availability in English. Grey literature (reports from UNESCO, UNCTAD, and the IOC) was included where directly relevant to the framework's empirical grounding. Approximately 40 sources were identified, reviewed, and synthesized.

The literature was organized into five thematic clusters following Torraco’s (13) guidance on integrative review structure: (1) impacts and leverage of sport-event tourism; (2) theories of creative tourism; (3) creative placemaking; (4) social sustainability in tourism; and (5) co-creation in tourism experiences. The CSETN framework was developed through an iterative process of inductive synthesis and deductive reasoning: first, key theoretical models were mapped across the five clusters; second, points of convergence and complementarity were identified; third, integration mechanisms were inductively derived from documented empirical precedents and theoretical propositions; and fourth, the framework components were refined through iterative comparison with the case evidence. As an opinion piece, this article prioritizes conceptual novelty and synthetic contribution over comprehensive systematic coverage (15).

## Theoretical foundations

### Event leverage and sport-event tourism

Chalip’s (16) event leverage framework represents the primary theoretical anchor for this article's sport-event tourism component. Chalip argued that the economic and social impacts of sport events are not inherent properties of the events themselves but are produced by deliberate activities constructed around them. This reframing—from impact measurement to leverage strategy—established what subsequent scholarship has called the ‘leveraging turn’ in sport-event impact studies (16, 17). Operationally, event leveraging involves the strategic deployment of complementary activities—such as cultural programming, business networking, and community engagement—that use the event as a catalyst rather than treating it as a self-contained product. This creates explicit conceptual space for creative interventions as strategic leverage tools, providing the CSETN's primary rationale for integrating creative placemaking within sport-event tourism planning.

The relevance of this framework is reinforced by the extensively documented ‘mega-event syndrome,’ characterized by cost overruns, community displacement, and exclusion from planning processes (4, 18), which has generated sustained calls for participatory and leverage-oriented approaches that distribute benefits more equitably.

### The creative tourism framework

Richards (6) synthesizes the Creative Tourism Framework from earlier definitional work by Richards and Raymond (5) and subsequent empirical studies across European and Asian contexts. The framework rests on three interlocking components. First, the content dimension identifies the specific creative domains through which tourist engagement occurs, including crafts, performing arts, culinary arts, digital media, and heritage interpretation. Second, the process dimension distinguishes passive creative consumption (attending a performance) from active creative participation (co-producing a craft object or contributing to a community mural), with higher levels of participation associated with deeper experience quality and stronger place attachment. Third, the context dimension recognizes that creative tourism is fundamentally embedded in place: the authenticity and meaning of creative experiences derive from their rootedness in specific communities, traditions, and physical environments. Applied to the CSETN, these three dimensions map directly onto the framework's six integration mechanisms: creative fan experiences and digital co-creation engage the content and process dimensions; artisan activations and co-designed event spaces engage the context dimension; cultural side-festivals and immersive storytelling engage all three simultaneously.

### Experience economy

Pine and Gilmore’s (19) Experience Economy framework posits that advanced economies increasingly compete on the basis of staged experiences rather than goods or services. Four realms of experience are identified—entertainment, educational, aesthetic, and escapist—distinguished by the degree of active participation (passive to active) and environmental immersion (absorption to immersion). The framework has been applied extensively in tourism contexts to explain value creation through experience staging. Within the CSETN, the Experience Economy provides the commercial rationale for creative integration: experiential differentiation increases visitor willingness to pay, extends dwell time, and enhances destination competitiveness.

However, a theoretical tension warrants explicit acknowledgment. The Experience Economy is fundamentally market-driven, framing experience as a staged commercial offering designed for and sold to consumers. Creative placemaking (10), by contrast, is community-oriented, prioritizing resident agency, cultural equity, and non-commercial meaning-making. The CSETN holds this tension productively rather than attempting to resolve it, recognizing that successful integration requires deliberate governance structures that prevent market logic from colonizing community-led creative processes. Where event organizers treat creative placemaking purely as a marketable differentiator, risks of authenticity loss and community alienation increase. Where community capacity and participatory governance are prioritized, the two frameworks can complement rather than contradict one another.

### Social exchange theory

Social Exchange Theory (SET (20); provides the micro-level foundation for understanding community–visitor relationships within creative interventions. SET posits that individuals and groups engage in social exchanges when perceived benefits are seen to outweigh costs. Applied to the CSETN, this explains why residents may actively support or resist creative programming associated with sport events. When communities perceive tangible benefits—income from artisan markets, cultural recognition through co-designed event spaces, pride generated by heritage storytelling installations—they are more likely to participate in and sustain creative initiatives. Conversely, where benefits accrue primarily to external operators rather than local residents, disengagement and resistance are predictable outcomes. SET thus provides the motivational logic underpinning the CSETN's emphasis on inclusive co-design and equitable benefit-sharing, and it connects directly to the social inclusion outcome pathway of the framework.

### Integration of theoretical foundations

These four theoretical frameworks are not independent pillars but interconnected contributors to the CSETN's explanatory architecture. The Event Leverage Framework (16) establishes *why* creative interventions are strategically valuable within sport-event tourism planning. The Creative Tourism Framework (6) specifies *what* forms creative tourism takes and *how* visitor engagement deepens across content, process, and context dimensions. The Experience Economy (19) explains the commercial *value* generated through experiential differentiation. Social Exchange Theory (20) provides the community-level logic that determines *whether* residents support or resist creative integration. Together, these frameworks form a multi-level explanatory system—from strategic event planning to visitor experience design to community participation dynamics—that the CSETN operationalizes through its six integration mechanisms.

## Synthesis of intersectional literature

As noted in the introduction, direct empirical engagement with the intersection of sport-event tourism, creative tourism, and creative placemaking remains sparse. Table 1 synthesizes the key studies identified through the scoping search, reporting their focus, methodology, key findings, and contribution to understanding the CSETN intersection.

**Table 1:** Synthesis of key studies at the intersection of sport-event tourism and creative tourism/placemaking.

Study	Focus	Method	Key findings	Contribution to CSETN
García (22)	Barcelona 1992 Cultural Olympiad	Documentary analysis, case study	Creative programming generated sustained destination repositioning; 65% increase in arrivals 1990–1995	Establishes cultural side-festival mechanism as empirically grounded
Smith (17)	London 2012 Cultural Olympiad	Case study, stakeholder interviews	Cultural leveraging strengthened community identity but required dedicated governance	Supports co-design mechanism; highlights governance moderating factor
Stokes (21)	Melbourne Grand Prix tourism strategy	Qualitative interviews, strategy analysis	Events generate creative economy activation opportunities when leveraging is intentional	Grounds creative fan experience mechanism in event strategy literature
Chalip (16)	Event leverage theory	Conceptual, literature review	Events create conditions for leveraged creative and social activation	Core theoretical foundation for all six nexus mechanisms
Ziakas & Costa (25)	Event portfolio and community development	Mixed methods, regional case	Co-designed event portfolios generate stronger community ownership	Grounds co-designed event spaces mechanism
Tan et al. (29)	Creative experience model in tourism	Survey-based, empirical	Co-creation, novelty, and self-expression produce deeper tourist experiences	Grounds visitor-side value creation logic of CSETN
Neuhofer et al. (26)	Technology-enhanced tourism experiences	Conceptual typology with cases	AR/VR and digital tools amplify co-creation and immersion	Grounds immersive storytelling and digital co-creation mechanisms
Duxbury & Richards (31)	Creative tourism research agenda	Literature review	Creative tourism intersects with placemaking through community-embedded practice	Positions creative placemaking as bridge concept
Lee & Preuss (23)	Olympic legacy measurement	Longitudinal case analysis	PyeongChang cultural programming generated short-term gains but limited durable legacy	Highlights event scale and post-event governance as moderating factors
Richards & Marques (24)	Creative tourism exploration	Comparative case studies	Creative tourism extends visitor dwell time and deepens local economic linkages	Supports artisan market mechanism and economic diversification outcome
Richards (6)	Creative tourism: state of the art	Integrative review	Creative tourism distinguished by active participation across content, process, context dimensions	Provides Creative Tourism Framework for CSETN
Smith (18)	Events and urban regeneration	Case studies	Event-led regeneration risks gentrification; community governance is protective	Grounds boundary conditions subsection
IOC (27)	Tokyo 2020 cultural programme	Institutional report	Digital creative programming extended participation globally beyond host city	Supports digital co-creation mechanism empirically
Markusen & Gadwa (10)	Creative placemaking	Policy review and cases	Arts integration into community life generates civic engagement and place identity	Core placemaking concept underpinning CSETN context dimension
Rihova et al. (7)	Customer-to-customer co-creation	Conceptual and empirical	Co-creation among visitors and communities generates emergent experiential value	Defines co-creation concept and its value logic within CSETN

Studies are drawn from the scoping search across Web of Science, Scopus, and Google Scholar (2000–2024). This table represents direct intersectional sources; additional sport-event tourism and creative tourism studies cited in the text provide broader theoretical grounding.

**Table 2:** Typology of creative interventions in sport-event tourism: destination value (DV), social impact (SI) contributions, and evidence level.

Intervention type	Description & examples	Destination value	Social impact	Evidence level
Creative Fan Zones & Interactive Installations	Participatory art walls, fan murals, interactive digital displays in event precincts	Experiential quality, brand differentiation, extended dwell time	Inclusive creative participation; community pride via local artist involvement	Emerging Evidence (17, 21)
Cultural Side-Festivals & Heritage Trails	Arts/music festivals, food trails, heritage tours parallel to sport events	Economic diversification, extended stay, cultural capital	Cultural preservation; equitable economic distribution to local businesses	Established Practice (16, 22)
Artisan Markets & Creative Workshops	Pop-up craft markets, hands-on workshops linked to event themes	Multiplier effects, experiential depth, repeat visitation	Direct income to local producers; marginalized community empowerment	Established Practice (23, 24)
Community Co-Designed Event Spaces	Residents and artists co-design fan zones, venue aesthetics, public art	Authentic place identity, distinctive visual branding	Anti-displacement; participatory governance; heritage preservation	Theoretical Proposal, with partial precedent (25, 31)
Immersive Storytelling & AR/VR	AR heritage overlays, athlete biography installations, destination mythology tours	Innovation capacity, memorability, digital brand extension	Multi-sensory accessibility; heritage interpretation for diverse audiences	Emerging Evidence (26)
Eco-Creative Installations	Upcycled infrastructure, nature-based art, environmental awareness installations	Green branding, innovation showcase	Environmental sustainability; community education	Theoretical Proposal
Digital Co-Creation Platforms	Social media challenges, collaborative digital art, gamified cultural apps	Pre/post-event engagement, user-generated content marketing	Global creative participation; remote accessibility	Emerging Evidence (26, 27)

Evidence levels classified as: Established practice=multiple studies with consistent empirical support; Emerging evidence=one to three empirical studies or documented outcomes; Theoretical proposal=conceptually grounded but lacking direct empirical support to date.

## The CSETN framework

### Framework development

The CSETN framework was constructed through the iterative synthesis process described in the methodology section, drawing directly on the intersectional literature synthesized in Table 1. In the inductive phase, recurring themes across the identified studies were mapped: the potential for sport events to activate creative economy supply chains; the role of community co-design in generating authentic place identity; and the governance conditions required for creative legacies to persist beyond the event period. In the deductive phase, these inductive themes were structured using the four theoretical frameworks described above—Event Leverage, Creative Tourism, Experience Economy, and Social Exchange Theory—to identify which mechanisms correspond to which theoretical logic. The six nexus mechanisms emerged from this dual process: each mechanism has both an empirical precedent in the intersectional literature (Table 1) and a theoretical grounding in one or more of the four frameworks. The dual outcome pathways—enhanced destination value and improved social inclusion—were derived deductively from the framework's theoretical architecture: destination value maps primarily to the Experience Economy and Event Leverage logics, while social inclusion maps to Social Exchange Theory and the Creative Tourism Framework's context dimension.

### Framework overview

**Figure 1:**
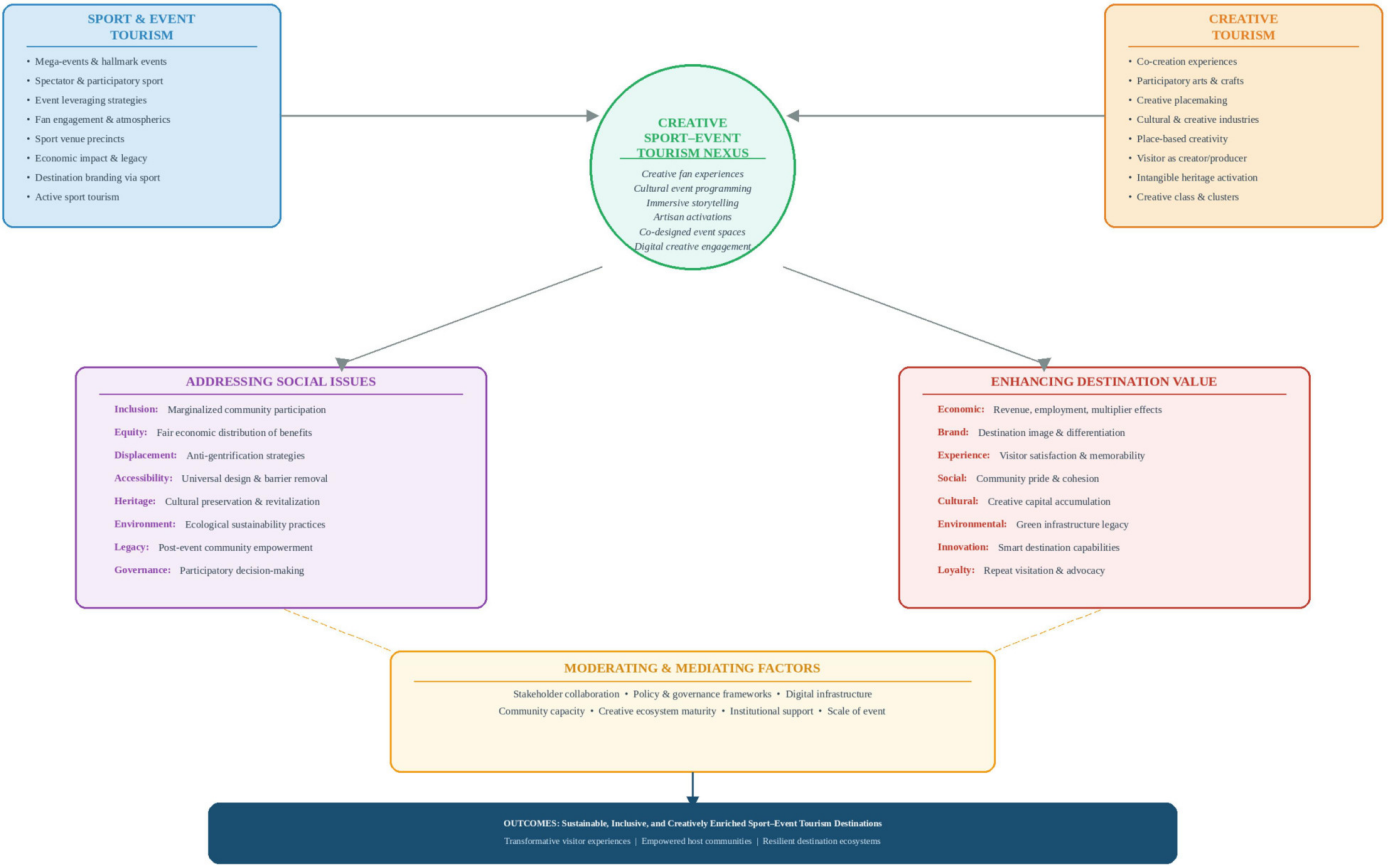
The Creative Sport–Event Tourism Nexus (CSETN) framework illustrating convergence mechanisms, dual outcome pathways, and moderating factors. Source: Authors’ own elaboration based on Richards (6), Chalip (16), Markusen and Gadwa (10), Pine and Gilmore (19), and Duxbury and Richards (31).

The Creative Sport-Event Tourism Nexus comprises five interconnected components (Figure 1): (1) sport-event tourism inputs—visitor concentration, media reach, and capital investment generated by the event; (2) creative tourism inputs—local creative assets, community participation capacity, and established cultural programming traditions; (3) the nexus domain where these converge through six integration mechanisms; (4) dual outcome pathways—enhanced destination value and addressed social inclusion challenges; and (5) moderating factors that shape the conditions under which mechanisms generate positive outcomes.

The five components operate in a bidirectional and iterative rather than strictly sequential manner. Sport-event inputs and creative tourism inputs feed simultaneously into the nexus domain; the resulting outcomes, in turn, generate feedback that reshapes subsequent inputs through learning, reputation-building, and community capacity development. This cyclical dynamic is consistent with Chalip’s (16) leverage framework, in which leveraging activities require ongoing refinement across event cycles.

### The Six nexus mechanisms

The six nexus mechanisms are differentiated below according to their level of empirical grounding: *Established Practice* (supported by multiple studies with consistent empirical evidence), *Emerging Evidence* (supported by one to three studies or documented outcomes), and *Theoretical Proposal* (conceptually grounded but lacking direct empirical support to date).

- **Creative fan experiences** [*Emerging Evidence*] engage spectators as participants through interactive installations and collaborative performances. Documented precedents include community murals and interactive digital precincts at the Melbourne Grand Prix and Commonwealth Games events, where participatory creative activations extended visitor engagement beyond the core sporting spectacle (17, 21). Theoretically, this mechanism aligns with Pine and Gilmore’s (19) escapist and educational experience realms, which require active participation.
- **Cultural side-festivals** [*Established Practice*] extend the event's reach through parallel programming such as arts festivals and heritage trails. The Barcelona Cultural Olympiad (1992) incorporated over 2,500 events across four years, contributing to a 65% increase in international arrivals between 1990 and 1995 (22). This mechanism has the strongest empirical base of the six, with consistent evidence from Olympic and hallmark event contexts (17, 22).
- **Artisan activations** [*Established Practice*] link local creative producers to the sport-event tourist market through pop-up craft markets and participatory workshops. The Gangwon-do regional programme associated with PyeongChang 2018 provided a platform for over 200 local producers to access the event visitor market, contributing to regional creative economy diversification (23). Richards and Marques (24) provide broader evidence for artisan activation as a mechanism for extending visitor spending within local supply chains.
- **Community co-designed event spaces** [*Theoretical Proposal, with partial precedent*] engage residents and artists in the aesthetics and functionality of fan zones and public art installations. While participatory design approaches have demonstrated potential to strengthen community ownership of event-linked spaces and reduce social opposition to event-related development (25), systematic empirical evidence from sport-event contexts specifically remains limited, qualifying this mechanism as a theoretical proposal supported by conceptual precedent rather than a tested intervention model.
- **Immersive storytelling** [*Emerging Evidence*] creates emotional connections between visitors and place through augmented reality heritage tours and narrative installations. The application of technology-enhanced experience design in event contexts is documented (26), though systematic outcome measurement remains nascent. This mechanism draws on Pine and Gilmore’s (19) aesthetics and escapist realms and Richards’s (6) content dimension of creative tourism.
- **Digital co-creation platforms** [*Emerging Evidence*] amplify participation through gamification and social media co-creation. The IOC's digital cultural programme at Tokyo 2020 demonstrated that digital co-creation can extend event participation to global audiences well beyond the physical host city (27). While the scale of participation is documented, causal effects on destination value and social inclusion require further empirical testing.

Table 2 provides a typology of these creative interventions, summarizing their destination value and social impact contributions alongside evidence levels.

### Moderating factors

Five moderating factors shape the conditions under which nexus mechanisms generate positive outcomes.

1. **Stakeholder engagement**, operationalized as the percentage of local residents and community organizations actively involved in co-design processes, determines the authenticity and legitimacy of creative interventions. Based on participatory governance literature (25), a minimum threshold of approximately 30% local stakeholder involvement in planning processes is proposed as an illustrative benchmark for meaningful co-governance; this figure is offered as a practical reference point for practitioners and researchers rather than a precisely evidence-calibrated threshold, and its empirical justification warrants formal testing in future research.
2. **Policy environment**, measured by the presence or absence of formal creative economy strategies and statutory event legacy planning requirements within the host jurisdiction, sets the regulatory and financial conditions for sustained integration.
3. **Digital infrastructure**, operationalized as broadband penetration rate and availability of AR/VR-capable platforms at event venues, determines the viability of technology-dependent mechanisms such as immersive storytelling and digital co-creation.
4. **Community creative capacity**, measured as the number of registered creative enterprises per 10,000 population and the presence of established arts and cultural organizations, determines whether sufficient local supply exists to populate artisan markets, co-design processes, and cultural programming.
5. **Event scale**, operationalized through total visitor numbers, broadcast audience reach, and event duration, determines both the resource envelope available for creative programming and the extent to which commercial pressures may override community-oriented objectives.

### Boundary conditions: negative synergies and risks

The CSETN does not operate uniformly across all contexts, and acknowledging its boundary conditions is essential for responsible application. The most significant risk concerns gentrification: creative placemaking activities associated with high-profile sport events can accelerate property value inflation, displacing the very communities whose cultural assets underpin the creative programming. This dynamic has been documented in Olympic host cities, where artistic and cultural regeneration of inner-city precincts preceded significant displacement of low-income residents (18). A second boundary risk is the commodification of local culture: when creative interventions are designed primarily to serve the experiential demands of visiting sport tourists, authentic cultural expression may be reduced to a performative spectacle that loses meaning for resident communities over time.

A broader concern is that mega-event sustainability and legacy commitments are frequently neither uniformly positive nor genuinely ‘win-win.’ Evidence from hotel sustainability research across five recent mega-event host cities documents recurring patterns of short-lived initiatives, cost burdens on local operators, and limited post-event accountability mechanisms, even where event-period sustainability commitments were substantial (28). Creative legacy investments face analogous structural challenges: without binding post-event governance frameworks, creative infrastructure risks becoming ‘event decoration’ rather than a foundation for durable placemaking.

Three structural conditions increase the probability of negative synergies. First, where event organizers retain primary control of creative programming budgets without formal community co-governance mechanisms, commercial priorities will tend to dominate. Second, where host cities lack rent stabilization or community benefit agreement frameworks, creative gentrification risks are amplified. Third, where the event is a one-time mega-event rather than an embedded recurring event, creative investments are less likely to produce sustainable community legacies. Practitioners applying the CSETN are urged to conduct community impact assessments prior to implementing creative interventions and to establish binding benefit-sharing agreements with local creative stakeholders.

## Discussion

### Converging pressures driving CSETN relevance

The rationale for integrating creative tourism with sport-event tourism lies in several converging pressures. Economically, the creative economy is among the fastest-growing sectors globally, with a valuation of USD 2.25 trillion in 2015 (9), and sport events provide a concentrated activation opportunity with high visitor density and media exposure. Experientially, travelers increasingly seek participation rather than passive spectatorship (7, 29). Socially, growing critiques of mega-event impacts create demand for approaches that prioritize local benefit distribution (4). Technologically, digital and immersive media make large-scale co-creative experiences feasible at event scale (26, 27).

### Case analysis: empirical illustration of CSETN dynamics

Three empirical cases are analyzed below to illustrate CSETN dynamics across varying event contexts, host capacities, and outcome profiles. Table 3 provides a structured comparative overview before each case is discussed analytically.

**Table 3:** Comparative case analysis: CSETN mechanisms and outcomes across three sport-event tourism contexts.

Dimension	Barcelona 1992 Cultural Olympiad	PyeongChang 2018 Winter Olympics	Tokyo 2020 Olympic Games
Event type	Summer Olympics (mega-event)	Winter Olympics (mega-event)	Summer Olympics (mega-event, digital-hybrid)
Host context	Major European city with established creative economy	Smaller regional city, South Korea	Major global metropolis, digital infrastructure leader
Primary CSETN mechanisms activated	Cultural side-festivals, co-designed spaces, artisan activations	Artisan activations, cultural side-festivals	Digital co-creation platforms, immersive storytelling
Destination value outcomes	65% increase in international arrivals (1990–1995); sustained repositioning as European tourism hub	Regional creative economy diversification; 200 + local producers accessed event market	Global audience engagement; digital brand extension beyond host city
Social inclusion outcomes	Cultural recognition through 4,000 + artist engagements; however, gentrification documented in surrounding districts	Income generation for regional producers; capacity limitations constrained broader community benefits	Digital participation expanded access globally; local community displacement risks lower due to existing infrastructure
Legacy durability	High — creative programming embedded in long-term urban development strategy	Low-to-moderate — post-event facility utilization fell below 30% within two years	Moderate — digital legacy assets remain accessible; physical legacy limited by pandemic context
Key moderating factors	Strong policy environment and community creative capacity; large event scale enabled substantial creative budget	Smaller host city with limited creative capacity; weak post-event governance frameworks	Strong digital infrastructure; advanced policy environment; unusual event context (COVID-19 restrictions)
Primary lesson for CSETN	Creative programming generates durable destination value when embedded in long-term urban strategy with strong governance	Event scale and community creative capacity are critical moderators; smaller host cities face structural disadvantages	Digital mechanisms can compensate for reduced physical access but require robust pre-event platform development

Source: Authors’ own elaboration based on García (22), Lee and Preuss (23), IOC (27), and Smith (17).

#### Barcelona 1992

The Barcelona Cultural Olympiad—the most extensively documented example of creative programming within a sport mega-event—engaged over 4,000 artists across approximately 2,500 events over four years and attracted an estimated 35 million cumulative attendances. Cultural investment contributed directly to Barcelona's transformation into one of Europe's leading city tourism destinations (22). The case illustrates CSETN dynamics under favorable conditions: a large, diverse host city with established creative industries, a strong policy environment that integrated Olympic planning with longer-term urban regeneration strategy, and substantial public investment that enabled sustained creative programming before, during, and after the event. The 65% increase in international arrivals between 1990 and 1995 cannot be attributed solely to creative programming—urban regeneration, infrastructure investment, and changing European travel patterns all contributed—but García’s (22) analysis establishes cultural programming as a significant contributing factor to destination repositioning. Critically, however, the case also illustrates the gentrification risk: creative-led regeneration of inner-city precincts preceded significant residential displacement in districts including Poblenou, demonstrating that positive destination value outcomes and negative social inclusion outcomes can co-occur without adequate housing and community benefit governance.

#### Pyeongchang 2018

The PyeongChang Winter Olympics incorporated over 300 cultural performances and invested approximately USD 100 million in cultural programming across the Cultural Olympiad. The Gangwon-do regional programme—aligned with CSETN's artisan activation and cultural side-festival mechanisms—provided over 200 local producers with access to the event visitor market (23). However, the case illustrates the constraining effects of two moderating factors: event scale and community creative capacity. As a smaller, less urbanized host city with a limited pre-existing creative sector, PyeongChang faced structural disadvantages in converting event-period creative activation into durable placemaking outcomes. Post-event facility utilization rates fell below 30% within two years, and several creative venues were decommissioned within three years of the Games (23). These difficulties parallel findings on hotel sustainability legacies, where event-time improvements frequently fail to translate into durable operational changes without robust post-event management frameworks (28). Both cases underscore that CSETN mechanisms require strong post-event governance frameworks to generate legacy outcomes.

#### Tokyo 2020

The Tokyo 2020 Games, conducted in a pandemic context that severely restricted physical attendance, demonstrate the CSETN's digital co-creation mechanism under unusual but analytically informative conditions. The IOC's digital cultural programme reached global audiences through digital exhibitions, collaborative online art projects, and gamified cultural engagement platforms, demonstrating that digital mechanisms can extend creative event participation beyond the physical host city (27). The Tokyo case adds an important dimension to the CSETN by illustrating that digital infrastructure moderates the accessibility of certain mechanisms: host cities with strong digital infrastructure can activate digital co-creation platforms as primary rather than supplementary mechanisms, potentially compensating for reduced physical creative programming capacity.

### Applicability in low- and middle-income country contexts

The transferability of the CSETN to low- and middle-income country (LMIC) contexts warrants specific and expanded consideration, given the increasing frequency of mega-events and major sport tournaments hosted in LMIC settings. While events such as the 2010 FIFA World Cup in South Africa have demonstrated the potential for associated cultural programming to stimulate local creative industries, the structural conditions in LMIC contexts differ substantially from those in high-income host cities and require tailored implementation approaches.

Resource constraints are the most immediate challenge. Capital-intensive creative interventions—purpose-built artisan market pavilions, venue-integrated AR infrastructure, large-scale co-designed public art commissions—may be financially inaccessible for host municipalities with limited event budgets and competing infrastructure priorities. In LMIC settings, CSETN implementation is more feasible through low-capital mechanisms: community-organized cultural performances, informal artisan market activations in existing public spaces, and mobile digital co-creation platforms that leverage existing smartphone penetration rather than requiring dedicated venue infrastructure.

Institutional constraints present a second significant challenge. LMIC host cities frequently lack the statutory event legacy planning frameworks, formal creative economy strategies, and inter-departmental coordination mechanisms that the CSETN's policy environment moderator identifies as enabling conditions. Capacity-building investments in institutional coordination and community creative enterprise development must therefore precede or accompany event-linked creative programming rather than assuming existing institutional capacity is available for activation. This mirrors findings on hotel sustainability in LMIC host cities, where organizational readiness significantly moderates the extent to which mega-event pressures translate into lasting sustainability improvements (28).

Heightened displacement risks represent a third structural concern specific to LMIC contexts. Where housing markets are weakly regulated and social protection systems are limited, the gentrification effects documented in high-income host cities may be more severe. Community co-design mechanisms and co-designed event spaces—the CSETN components with the strongest anti-displacement logic—should therefore be prioritized in LMIC implementation, even though they currently represent the weakest empirical foundation of the six mechanisms. Mandatory community impact assessments and binding benefit-sharing agreements with local creative stakeholders are particularly important in these contexts.

Finally, the community creative capacity moderator takes on heightened importance in LMIC settings. Formal creative enterprise infrastructure may be limited or informally organized, and existing cultural traditions may be embedded in community practice rather than commercially registered entities. CSETN implementation in LMIC contexts should recognize informal creative economy actors as legitimate stakeholders and design activation mechanisms that are accessible to informal producers, traditional artisans, and community cultural organizations rather than relying exclusively on formally registered creative enterprises.

### Implementation barriers

Barriers to CSETN integration should not be minimized. Institutional silos between event management, cultural policy, and tourism promotion impede coordinated delivery. Compressed event planning timelines disadvantage participatory co-design processes that require sustained community engagement over months or years. Fragmented funding streams create accountability gaps between event sponsors, public funders, and community organizations. Performance measurement frameworks that prioritize economic over social or cultural outcomes render creative programming investments difficult to justify within conventional event reporting structures (30). Addressing these barriers requires treating the sport event not merely as a time-bounded economic opportunity but as a catalyst for longer-term creative destination development.

## Research agenda and policy implications

### Prioritized research agenda

The following five research priorities are ordered by feasibility, from near-term achievable to longer-term and resource-intensive.

1. **Developing composite measurement indicators (immediate feasibility).** The most pressing near-term need is the development of validated composite indicators capable of measuring social, cultural, and creative value in sport-event tourism contexts. Existing economic impact tools are well established; social and cultural equivalents remain fragmented. Survey-based instruments adapted from creative economy metrics (9) and social sustainability indices represent a tractable starting point.
2. **Multiple-case empirical testing of CSETN mechanisms (medium-term feasibility).** Comparative case studies across diverse event types (mega-events, hallmark events, community sport events) and geographical contexts would provide the empirical grounding currently absent from the framework. Case selection should deliberately include LMIC settings to test transferability assumptions and refine the 30% stakeholder engagement threshold proposed in the moderating factors section.
3. **Quasi-experimental comparison of co-creative vs. passive event experiences (medium-term feasibility).** Controlled comparisons between events that integrate community co-creative programming and those that do not—matched on event scale, host context, and visitor demographics—would strengthen causal inference regarding the value-creation potential of CSETN mechanisms.
4. **Longitudinal legacy tracking (long-term feasibility).** Multi-year tracking of creative programming initiatives across event cycles is necessary to measure creative placemaking's contribution to post-event legacies. Research designs should follow communities and creative organizations across a minimum of five years post-event.
5. **Participatory action research with marginalized communities (long-term, resource-intensive).** The most complex priority involves employing participatory action research methodologies to co-design inclusive creative interventions with groups most at risk of displacement or exclusion. This requires sustained community engagement, ethical oversight, and longitudinal commitment that goes beyond conventional academic project timescales.

### Stakeholder-differentiated policy implications

**Event organizers** should establish minimum budget thresholds for community-led creative programming—a recommended allocation of at least 5% of total event operational budgets dedicated to locally sourced creative initiatives. Procurement processes should require documented evidence of community co-design participation. Event master plans should include a dedicated creative legacy chapter specifying how creative infrastructure and community capacity built during the event period will be sustained beyond event conclusion.

**Municipal governments** should adopt co-design procurement standards requiring meaningful local artist and community organization involvement in the commissioning of event public art, fan zone design, and cultural side-programming. Pre-event community impact assessments should be mandatory for events exceeding a defined visitor threshold, with findings incorporated into event licensing conditions. Municipalities should establish inter-departmental coordination mechanisms linking event management, cultural policy, and urban planning to overcome the institutional silos that currently impede integrated CSETN delivery.

**National tourism boards** should develop social impact key performance indicators (KPIs) for event-linked creative tourism, including cultural participation rates among host community residents, percentage of creative economy revenue retained by local businesses, resident satisfaction with event-related creative programming, and post-event creative enterprise formation rates. These KPIs should be incorporated into national event bidding strategies and reported alongside conventional economic impact metrics in post-event evaluation frameworks.

## Conclusion

Sport-event tourism stands at a creative crossroads. The CSETN framework proposed in this article identifies six specific integration mechanisms—grounded in four complementary theoretical frameworks and synthesized from the available intersectional literature—through which creative tourism concepts can be deployed within sport-event tourism contexts to enhance destination value and improve social inclusion simultaneously.

Three findings merit particular emphasis. First, the empirical grounding of the six mechanisms is uneven: cultural side-festivals and artisan activations rest on an established empirical base, while community co-designed event spaces and eco-creative installations remain largely theoretical proposals requiring dedicated empirical testing. This distinction is important for practitioners prioritizing mechanism selection and for researchers identifying the highest-value empirical contributions. Second, moderating factors—particularly community creative capacity and post-event governance frameworks—are decisive: CSETN mechanisms cannot be assumed to generate positive outcomes independently of context. Third, the framework's applicability in LMIC contexts requires scaled-down, community-led, and institutionally supported implementation rather than direct transfer of capital-intensive models from high-income host city contexts.

The theoretical tension between market-driven experience staging and community-oriented placemaking is not resolved by the CSETN but is held productively as a governance design challenge. Ensuring that creative placemaking functions as a foundation for durable community benefit—rather than as an event marketing device—requires deliberate institutional design, binding benefit-sharing arrangements, and post-event accountability mechanisms that practitioners must address at the outset of event planning.

The creative economy offers the tools and imagination; sport-event tourism provides the stage, the audience, and the urgency. The challenge now is to embed creative placemaking not as an afterthought, but as a foundational principle in the design, delivery, and evaluation of sport-event tourism in the twenty-first century.
